# EHMTI-0240. Modulation of central pain processing by anodal direct current stimulation – a fMRI study

**DOI:** 10.1186/1129-2377-15-S1-E20

**Published:** 2014-09-18

**Authors:** S Naegel, J Biermann, M Obermann, N Theysohn, HC Diener, D Holle

**Affiliations:** 1 Dept. of Neurology, University Hospital Essen, Essen, Germany; 2 Institute for diagnostic and interventional neuroradiology, University Hospital Essen, Essen, Germany

## Introduction

Drugtherapy of pain and headache disorders was substantially improved during the last decades. However, in some patients headaches poorly respond to drug treatment, especially in chronic courses of disease. Transcranial direct current stimulation (tDCS) offers a new therapeutic approach in these patients. The underlying mechanisms of this neuromodulatory method are poorly understood.

## Aims

To expand understanding of the underlying mechanism of the antinociceptive effect of anodal tDCS in healthy volunteers using functional magnetic resonance imaging.

## Methods

Thirteen subjects were investigated after left sided anodal-DC and sham stimulation. A fMRI block paradigm was used testing trigeminal nociceptive processing as well as visual (checkerboard) and motor-function (fingertapping) as control conditions. Painful trigeminal stimuli were applied using a specialized electrode highly specific for nociception.

## Results

The pattern of cerebral activation observed in single-subject and group-analyses were consistent with those previously reported for pain-, motor- and visual-processing. The analyses comparing sham and anodal-DC stimulation was not able to detect differences in cerebral motor or visual processing after stimulation, but revealed a significantly increased nociceptive activation in the middle temporal lobe and the ipsilateral hippocampus after anodal DC-stimulation.

## Conclusions

These preliminary results demonstrate a DC-dependend modulation of trigeminal pain processing resulting in an increased activation of the ipsilateral middle temporal lobe and the hippocampus after anodal stimulation. This altered activation pattern may represent the correlate of antinociceptive effects of this method, which may be based on increased antinociceptive activity of the middle temporal lobe and modified evaluation of pain in the hippocampus.

No conflict of interest.

